# LIFE AFTER 100: IS GOD THE SECRET TO A SATISFYING AND PURPOSEFUL LIFE?

**DOI:** 10.1093/geroni/igz038.1931

**Published:** 2019-11-08

**Authors:** Nadia Firdauysa, Jyoti Bhatta, Alex J Bishop, Tanya Finchum, James Grice

**Affiliations:** 1 Oklahoma State University, Stillwater, Oklahoma, United States; 2 Oklahoma Oral History Research Program, Oklahoma State University; Stillwater, Oklahoma, United States

## Abstract

Data from N = 111 centenarians (M = 100.88; SD = 1.48) residing in Oklahoma was used to examine patterns in the relationship between the God oriented vs. non-God oriented longevity secrets and subjective well-being. Observational Oriented Modeling (OOM) was then used to conduct an ordinal analysis using concatenated ordering to produce degree of fitness between data and underlying patterns in life satisfaction and purpose-in-life across three time points. OOM is a data analysis method used to evaluate fitness of proposed patterns to data called PCC. Results indicated that centenarians maintaining a God-oriented longevity secret fit a decreased pattern in life satisfaction (PCC = 25.00, c-value = .09); whereas centenarians not maintaining a God-oriented longevity secret fit the same pattern (PCC = 49.18, c-value = .06). Meanwhile, centenarians having a God-oriented longevity secret fit a decreased pattern of purpose-in-life (PCC = 71.43, c-value =.12); whereas centenarians having a non-God oriented longevity secret fit the same pattern (PCC = 53.45, c-value = .28). In comparison to centenarians who acknowledged something other than God as the secret to their longevity, those who cite God as the reason for longevity tend to proportionately maintain a more satisfying view of life, yet experience a deteriorating sense of purpose over time. Results indicate that longevity secrets reflect divergent patterns in subjective well-being among persons living beyond 100 years. This has implications relative to how geriatric practitioners design interventions, services, or programs to enhance quality-of-life for long-lived adults.

